# GAPDH in neuroblastoma: Functions in metabolism and survival

**DOI:** 10.3389/fonc.2022.979683

**Published:** 2022-10-04

**Authors:** Kevin Cornett, Anna Puderbaugh, Olivia Back, Rolf Craven

**Affiliations:** Department of Pharmacology and Nutritional Sciences, College of Medicine, University of Kentucky, Lexington, KY, United States

**Keywords:** neuroblastoma, metabolism, glycolysis, glucose, autophagy

## Abstract

Neuroblastoma is a pediatric cancer of neural crest cells. It develops most frequently in nerve cells around the adrenal gland, although other locations are possible. Neuroblastomas rely on glycolysis as a source of energy and metabolites, and the enzymes that catalyze glycolysis are potential therapeutic targets for neuroblastoma. Furthermore, glycolysis provides a protective function against DNA damage, and there is evidence that glycolysis inhibitors may improve outcomes from other cancer treatments. This mini-review will focus on glyceraldehyde 3-phosphate dehydrogenase (GAPDH), one of the central enzymes in glycolysis. GAPDH has a key role in metabolism, catalyzing the sixth step in glycolysis and generating NADH. GAPDH also has a surprisingly diverse number of localizations, including the nucleus, where it performs multiple functions, and the plasma membrane. One membrane-associated function of GAPDH is stimulating glucose uptake, consistent with a role for GAPDH in energy and metabolite production. The plasma membrane localization of GAPDH and its role in glucose uptake have been verified in neuroblastoma. Membrane-associated GAPDH also participates in iron uptake, although this has not been tested in neuroblastoma. Finally, GAPDH activates autophagy through a nuclear complex with Sirtuin. This review will discuss these activities and their potential role in cancer metabolism, treatment and drug resistance.

## Introduction

Neuroblastoma is a cancer of neural crest cells and is the most common tumor before the age of one. Although neuroblastoma accounts for 5% of pediatric cancers, it results in 9% of pediatric cancer deaths. Neuroblastoma is a remarkably heterogeneous disease in which some patients undergo spontaneous remission, while other patients endure disease progression that is largely refractory to treatment. Neuroblastomas arise from the neural crest sympathoadrenal lineage that would normally form sympathetic ganglia.

Disease progression is associated with amplification of the *MYCN* transcription factor in 20-30% of neuroblastomas (1). N-MYC is a basic helix-loop-helix protein that drives the expression of a number of genes associated with survival and proliferation (2), including genes that are required for glycolysis (3, 4). This review will focus on the role of the GAPDH protein in metabolism, nutrient transport and survival in neuroblastoma.

## Glycolysis and cancer

Non-malignant human cells release energy *via* oxidative phosphorylation, which is highly efficient, and use other less efficient pathways like glycolysis under specific stress conditions. However, cancer cells prefer to utilize glycolysis, producing lactate and pyruvate, even when oxygen is present. This metabolic preference, called the Warburg Effect (5), is a central property of cancer cells and is the target of a number of emerging therapeutic approaches. In neuroblastoma, the activity of succinate dehydrogenase is significantly reduced (6), reflecting the shift to glycolysis.

Glycolysis is not as efficient in producing energy as oxidative phosphorylation, producing a profound requirement for glucose uptake that has been a mainstay of cancer imaging for decades. Although glycolysis is not particularly efficient in generating ATP, it produces metabolic intermediates that include precursors of nucleotides, amino acids and lipids that are needed in rapidly proliferating cells, and these metabolites have been reviewed (7, 8). Cancer cells preferentially utilize anaerobic glycolysis, generating lactate, and rely on glutamine metabolism for metabolites (9). Some of the key glycolytic genes are regulated by pathways that are frequently altered in neuroblastoma (3), as are pathways associated with glutaminolysis (10, 11).

Importantly, tumor metabolism increases chemo- and radio-resistance (12, 13). A number of studies suggest that inhibition of glycolysis is effective against multiple types of cancer (14–19). Neuroblastomas have high levels of glucose uptake, high lactic acid production and low oxygen consumption (3), and there is evidence that glycolysis plays an important role in clinical responses in the disease. Doxorubicin is a standard chemotherapeutic agent used in the treatment of intermediate and high-risk neuroblastoma. When doxorubicin-resistant neuroblastoma cells were treated with both a glycolysis inhibitor and doxorubicin, cell viability was significantly decreased compared to either treatment alone, suggesting that glycolysis may increase chemoresistance (20).

One mechanism of chemoresistance involves P-glycoprotein, a drug efflux pump that relies on ATP. Decreased chemoresistance in the presence of a glycolysis inhibitor could possibly be explained by compromised P-glycoprotein activity due to ATP depletion following glycolysis inhibition (20). However, several studies suggested that the role and expression of P-glycoprotein in neuroblastoma cells remain to be fully elucidated (21, 22).

## Background on GAPDH

GAPDH catalyzes the fifth step in glycolysis, the conversion of glyceraldehyde 3-phosphate into 1,3-biphosphoglycerate (23), a reaction that generates NADH- a “high energy” compound that subsequently generates ATP. GAPDH is best known for this reaction and is often used as a “housekeeping” gene or loading control in expression analyses, belying its complex role in growth and survival. GAPDH contains two domains- for NAD^+^ binding and the catalytic domain. At the junction, cysteine 149 is a site for post-translational modification and is required for multiple GAPDH functions.

While GAPDH is generally localized to the cytoplasm, it’s sub-cellular localization can change, particularly when modified by nitrosylation, oxidation and phosphorylation (23). This review will focus on three areas as they pertain to neuroblastoma: (i) the role of GAPDH in glycolysis, (ii) GAPDH functions in plasma membrane nutrient transport, and (iii) GAPDH nuclear functions as they pertain to DNA repair and the control of apoptosis and autophagy. Other functions of GAPDH, including RNA binding and tRNA nuclear export have been reviewed elsewhere (23).

## GAPDH at the plasma membrane

A subset of GAPDH has been identified at the plasma membrane (23), and we detected plasma membrane GAPDH in neuroblastoma cells by membrane labeling (24). In other cell types, GAPDH at the plasma membrane catalyzes membrane fusion (25) and acts as a receptor for iron carrier proteins (26, 27). The role of the former in neuroblastoma is unclear. However, iron is increasingly linked to neuroblastoma, because there is an elevated requirement for iron in the disease (28, 29), and because there is an emerging link between MYCN and ferroptosis- a type of cell death mediated by iron (30). However, it is unclear whether GAPDH-mediated iron uptake has any role in ferroptosis in neuroblastoma. Membrane GAPDH also acts as a plasminogen receptor in infiltrating macrophages (31), and it is intriguing to consider that cancer cells might utilize this function to promote invasion. However, this idea is purely speculative.

**Figure 1:**
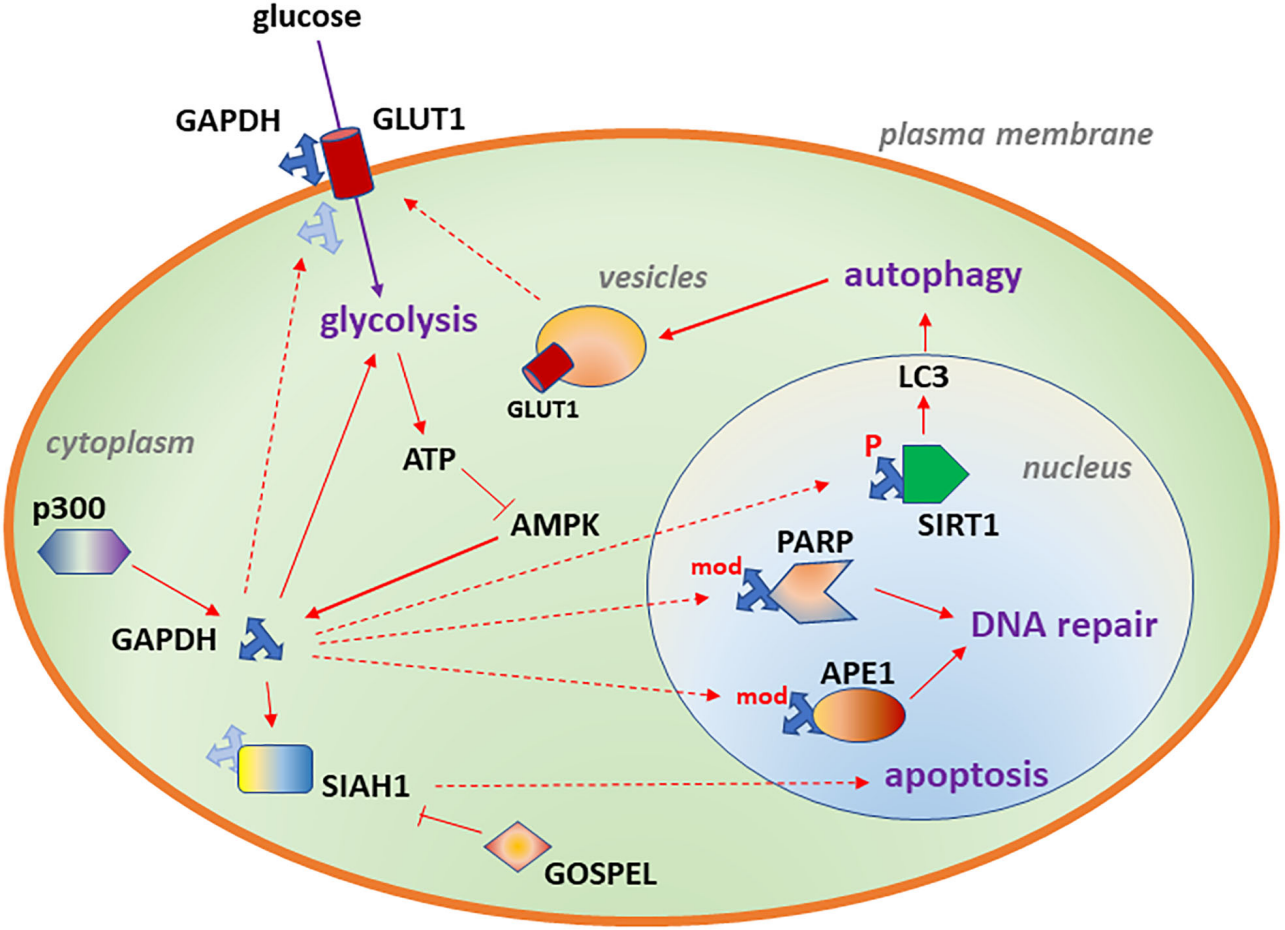
Diagram depicting subcellular localizations of GAPDH associated with glycolysis (cytoplasm), DNA repair (nucleus), autophagy (nucleus) and increased glucose transport (plasma membrane). Likely binding partners are also shown. Details of the model are described in Aim 1.

In neuroblastoma cells, GAPDH contributes to glucose uptake (24). This function resembles earlier findings in which GAPDH physically associates with GLUT1 in erythrocyte membranes (32) (**Figure 1**). Similarly, GAPDH associates with the intracellular surface of GLUT4 in rat L6 myotubes (33), and GAPDH expression is required for insulin-induced glucose transport. Glucose uptake is important in neuroblastoma, being used in imaging (34), and GLUT1 is a predictor of poor survival (35, 36). Furthermore, GLUT1 is essential for neuroblastoma cell survival in culture (36–38).

One model suggests that GAPDH binds to microtubules and localizes to secretory vesicles (39). The GAPDH-Pro234Ser mutant, which was identified by a chemical mutagenesis screen, inhibits vesicle transport in normal kidney cells (40). In this assay, GAPDH also bound to the AKT serine-threonine kinase- a key signaling protein in cancer cell survival. This assay utilized a purified form of wild-type or mutant GAPDH added directly to cells (40), suggesting an uncharacterized uptake pathway for the protein. It is intriguing to speculate that GAPDH’s putative function in binding AKT and vesicle trafficking might be related to the transport of metabolic proteins such as GLUT1 and GLUT4, but this function is uncharacterized in cancer cells. This model is supported by a role for GAPDH in vesicle biogenesis (41), vesicular glycolysis (42, 43), and inhibiting vesicle transport (44) but the relevance of these findings for neuroblastoma is not known.

## Nuclear functions of GAPDH

Upon modification, GAPDH can change binding partners and translocate to the nucleus, where it regulates multiple functions, including DNA repair. A number of modifications can cause GAPDH to translocate to the nucleus. These include phosphorylation of GAPDH-Ser122 by AMPK, AMP-dependent protein kinase (45). In contrast, AKT2 phosphorylation prevents nuclear translocation of GAPDH (46). The many chemical modifications of cysteine that alter GAPDH localization and alter its function have been reviewed recently (47). There are interactions between phosphorylation and other modifications, for example that AMPK phosphorylation can override nitrosylation (48), so that GAPDH-Ser122Ala mutants do not translocate to the nucleus (48), even if the protein undergoes other modifications.

In addition, GAPDH associates with the acetyltransferase p300/CBP (cyclic adenosine monophosphate response element/CREB-binding protein), which acetylates GAPDH on multiple lysine residues, which are required for translocation of a GAPDH-SIAH1 (seven in absentia homolog 1 E3 ubiquitin ligase) complex (49) to the nucleus (50). A number of studies of non-malignant tissues suggest that the transit of GAPDH to the nucleus after DNA damage promotes apoptosis (51–53). If GAPDH promotes apoptosis, then GAPDH inhibitors might suppress apoptosis in non-malignant cells while promoting cell death in cancer cells- a potential therapeutic window. Indeed, binding of GAPDH to the cytoplasmic protein GOSPEL (GAPDH competitor of SIAH protein enhances life/Rab interacting lysosomal protein like 1) competes with SIAH for binding to GAPDH and prevents NMDA-glutamate excitotoxicity (54). However, in some settings, nuclear GAPDH can perform repair or metabolic functions, and these are described next.

## GAPDH and DNA repair

Nuclear GAPDH contributes to DNA repair by binding DNA directly and enzymatically modifying DNA. Uracil is sometimes mis-incorporated into DNA as dUMP or is created through deamination of cytosine, and uracil-DNA glycosylase activity eliminates uracil from DNA and initiates the base excision repair pathway. GAPDH is a uracil-DNA glycosylase, and GAPDH also interacts with the repair protein APE1 [apurinic/apyrimidinic endodeoxyribonuclease 1 (55)], an endonuclease that creates a nick in the DNA phosphodiesterase backbone following the removal of a damaged base by a DNA glycosylase. GAPDH requires Cys152 for APE1 binding (55), and the GAPDH inhibitor koningic acid blocks active site cysteines of GAPDH, disrupting the GAPDH-APE1 interaction (55). As a result, koningic acid enhances the toxicity of H_2_O_2_-mediated oxidative damage in smooth muscle cells (55). Koningic acid was isolated from three different fungi from soil (56) and inhibits GAPDH (57) with resulting activity towards a variety of cancer cell types (58–60), including neuroblastoma (61). In some cases, koningic acid reverses drug resistance in cancer (62).

In addition to APE1 (apurinic/apyrimidinic endo-deoxyribonuclease 1), GAPDH also associates with the DNA repair protein PARP1, polyADP ribose polymerase (55, 63, 64), which is critical for single-strand break repair and double-strand repair (65). PARP can ADP-ribosylate and inactivate GAPDH, contributing to GAPDH depletion and in some cases, cell death (64, 66–68). Thus, mutants disrupting this interaction can elevate DNA damage and cell death through a failure in repair (55) or can inflict damage through uncontrolled PARP activity (64). Interestingly, some natural products have PARP inhibitory activity and may partially reverse this pathway (69).

## GAPDH and autophagy

Cancer cells activate numerous signaling pathways, particularly the mTOR (mammalian target of rapamycin) kinase, to inhibit autophagy (70). Numerous signaling, transport and proteolytic steps are in turn controlled by autophagy (71). In glioblastoma, GAPDH is included in an autophagy-related group of seven genes that served as a prognostic indicator (72). But autophagy has a complex role in neuroblastoma, as it does in other cancer types. Rapamycin inhibits mTOR and induces autophagy, and rapamycin induces growth arrest in neuroblastoma cells (73), suggesting a growth inhibitory role for autophagy in neuroblastoma. However, proteins that activate autophagy correlate with poor prognosis (70).

One possible explanation is that autophagy inhibits proliferation in the absence of cell stress but is necessary for cell survival under stressful or damaging conditions. Indeed, autophagy is associated with resistance to some types of chemotherapy [vincristine, doxorubicin and cisplatin (70)], endoplasmic reticulum stress (74), rotenone (75) and retinoid/apigenin treatment (76) in neuroblastoma. In contrast, autophagy uenhances or is required for cell death in neuroblastoma cells treated with the ribonuclease Onconase (77) the Akt inhibitor MK-2206 (78) and agents inducing oxidative damage (79). There are numerous additional examples of autophagy enhancing or inhibiting cell death in neuroblastoma, and some have been recently reviewed (63). Thus, the cell survival function of autophagy in neuroblastoma is dependent on the type of stress and precise experimental conditions (i.e. media components, time of treatment and experimental endpoints).

Given its role in energy metabolism, it is not surprising that GAPDH contributes to autophagy. Under low glucose conditions, ATP decreases and AMP increases, activating AMPK, which phosphorylates GAPDH on serine 122, triggering the translocation of GAPDH to the nucleus (48). There, GAPDH activates SIRT1/sirtuin 1, an NAD-dependent protein deacetylase, displacing a SIRT1 inhibitor and forming an active complex. Activated SIRT1, in turn, deacetylates LC3, a key marker of autophagy (80), increasing LC3 puncta formation in the cytoplasm, promoting autophagy (48), shown in **Figure 1**. These experiments were performed in mouse embryonic fibroblasts and HEK293 human embryonic kidney cells. In human pancreatic cancer cells, GAPDH nuclear translocation was inhibited by an arginine-273-histidine mutated form of the p53 tumor suppressor protein, favoring glycolysis and cell survival (63).

## GAPDH function in neuroblastoma

One of the key questions is how the different pathways directed by GAPDH might intersect in neuroblastoma. There are clues from the literature, although each has caveats. First, there are numerous gene expression studies in neuroblastoma using GAPDH as a loading control for other genes (81), in spite of concerns over utilizing GAPDH as a loading control (82). Other studies directly addressed the function of GAPDH in neuroblastoma. In cultured NG108-15 neuroblastoma-glioma hybrid cells, GAPDH inhibition for 24 hours efficiently induced apoptosis (61). Conversely, GAPDH was detected on the surface of Neuro2A and B103 neuroblastoma cells, and extracellular GAPDH increased neurite outgrowth in cultured neurons (83), although its role in neuroblastoma growth and survival were not determined.

A number of investigators have linked chemically modified forms of GAPDH to increased cell death (66, 84). Other studies support a role for nuclear GAPDH in the progression of apoptosis due to multiple stimuli in neuroblastoma (85–92). Aggregation of GAPDH into multimers also contributes to nuclear translocation and apoptosis (93, 94). Finally, secreted GAPDH may also contribute to cell death (95).

SHSY5Y cells are a widely used human model system that were derived from a 4-year-old girl with neuroblastoma. In studies with SHSY5Y cells, GAPDH inhibition with CGP3466B or heptilidic acid did not inhibit the formation of LC3-II (24), the cleaved and lipidated form of LC3 (80), but suppressed the degradation of the autophagosome target p62/SQSTM1, suggesting that GAPDH is not needed for the signaling pathways that initiate autophagy but is needed autophagic degradation in neuroblastoma. This is different from the GAPDH function in activating SIRT1 to promote LC3-II production (48), perhaps due to differences in glucose in the media. A separate study by Dodson, et al. in SHSY5Y cells after koningic acid (another name for heptilidic acid) treatment found minimal changes in LC3A-II and a slight increase in p62/SQSTM1 levels (96). But the conditions in the two studies were different. Craven, et al. used serum-free media for treatment (with high levels of autophagy), whereas Dodson, et al. used differentiated cells grown in 10% serum (with low levels of autophagy) and retinoic acid. It may be that high levels of autophagy detected in low serum were necessary to detect the effects of GAPDH inhibition. In the study by Dodson, et al., short-term (2 hour) GAPDH inhibition did not affect cell viability (96), and sensitivity to damaging agents such as chemotherapy was not measured. In that system, autophagy is required for viability when glycolysis is disrupted, underscoring a close and complex relationship between the pathways (96).

In a recent study, Ping, et al. used SHSY5Y cells in a co-culture system with *E. coli* expressing the α-synuclein, a soluble protein that regulates vesicle trafficking and neurotransmitter release. In this setting, GAPDH suppressed autophagy and promoted reactive oxygen species generation and cell death (97). However, this *E. coli* co-culture system expressing α-synuclein is not directly comparable to other systems. Finally, other investigators suggest an essential role for glycolysis in cell survival in SHSY5Y and SK-N-SH cells (98), based on the ability of ascorbate to induce cell death while suppressing glycolysis and inhibiting GAPDH activity. However, ascorbate also induced high levels of oxidative damage, suggesting that the effects of ascorbate were not attributable to a single mechanism.

## Discussion

GAPDH is a multi-functional protein with separate and antagonistic roles in cancer cell survival. While GAPDH-mediated glycolysis and DNA repair promote tumor cell survival, particularly in the presence of damage, GAPDH also has pro-apoptotic functions in response to different stimuli. Some of these functions are driven by changes in GAPDH cellular localization, and some GAPDH inhibitors are capable of blocking specific localizations. One factor that is less clear is how the function of GAPDH within the nucleus, for example, is directed towards repair versus pro-apoptotic functions or complexes that promote autophagy. Presumably, the availability of binding sites on partner proteins within the nucleus plays an important role, and this may be dynamic, depending on the quantity of any damage or metabolic stress that cells are experiencing.

One limitation to the use of GAPDH inhibitors for treating neuroblastoma is the possibility that some inhibitors that block GAPDH activity in glycolysis and glucose uptake may also inhibit apoptosis by suppressing a pro-apoptotic nuclear form of GAPDH. These would be contra-indicated when combined with chemotherapy. If these competing roles can be selectively inhibited, GAPDH has potential as a therapeutic target in neuroblastoma, particularly in combining GAPDH glycolytic, autophagy or DNA repair inhibitors with standard chemotherapy. For example, an ideal GAPDH inhibitor may inhibit the cytoplasmic and plasma membrane functions of GAPDH while leaving the pro-apoptotic nuclear function undisturbed. Other ideal inhibitors might selectively target nuclear GAPDH functions promoting autophagy or DNA repair while minimally inhibiting GAPDH pro-apoptotic activity. However, research in this area, including therapeutic development, is needed in order to advance GAPDH inhibitors as therapeutic approaches for neuroblastoma.

## Author contributions

KC, AP, and OB completed this paper as part of a Master’s thesis project (KC) or as a research project for the Honor’s College at the University of Kentucky (AP and OB). Each author contributed literature searches, article critiques and original text to the paper. RC assembled the manuscript, directed the research and wrote the majority of the paper. All authors read and approved the final manuscript.

## Funding

This research was funded in part by a University of Kentucky Department of Pharmacology and Nutritional Sciences Department Reinvestment Fund Award.

## Conflict of interest

The authors declare that the research was conducted in the absence of any commercial or financial relationships that could be construed as a potential conflict of interest.

## Publisher’s note

All claims expressed in this article are solely those of the authors and do not necessarily represent those of their affiliated organizations, or those of the publisher, the editors and the reviewers. Any product that may be evaluated in this article, or claim that may be made by its manufacturer, is not guaranteed or endorsed by the publisher.
